# Plasma S-Klotho level affects the risk of hyperuricemia in the middle-aged and elderly people

**DOI:** 10.1186/s40001-022-00875-w

**Published:** 2022-11-21

**Authors:** Haitao Xie, Ning Li, Guowei Zhou, Qian Liu, Haiyan Wang, Jie Han, Le Shen, Peng Yu, Jiandong Chen, Xiaohu Chen

**Affiliations:** 1 Department of Cardiology, Affiliated Hospital of Nanjing University of Chinese Medicine, Nanjing, China; 2 Department of Cardiology, Jiangsu Province Hospital of Chinese Medicine, No. 155, Hanzhong Road, Nanjing, 210004 China; 3 First Clinical Medical College, Nanjing University of Chinese Medicine, Nanjing, China

**Keywords:** S-Klotho, Aging, Hyperuricemia, Serum uric acid, NHANES

## Abstract

**Background:**

Soluble Klotho (S-Klotho) is an anti-aging protein mainly secreted by the kidneys. Hyperuricemia is prevalent among middle-aged and elderly individuals, which affects the development of various chronic diseases. However, there are relatively few studies investigating the association between plasma S-Klotho levels and hyperuricemia in middle-aged and elderly individuals. This study sought to clarify the relationship between S-Klotho and the risk of hyperuricemia in middle-aged and elderly people.

**Methods:**

During 2007–2016, a total of 50,588 people participated in the National Health and Nutrition Examination Survey. Finally, 12,441 middle-aged and elderly people (aged 40–79) completed the soluble Klotho tests and had obtained complete data. S-Klotho was detected by ELISA kit, and the relationship between S-Klotho and hyperuricemia was assessed by multiple logistic regression. Hyperuricemia is defined as serum uric acid levels higher than or equal to 420 mmol/l in men and 360 mmol/l in women.

**Results:**

In the middle-aged and elderly, plasma S-Klotho levels were negatively correlated with hyperuricemia, and there was a saturation effect. The inflection point of S-Klotho was 927.8 pg/ml (logarithmic likelihood ratio test = 0.002). When plasma S-Klotho < 927.8 pg/ml, the prevalence of hyperuricemia in middle-aged and elderly individuals with higher levels of S-Klotho decreased by 25.6% compared with those with low levels of S-Klotho [Q4 vs Q1, OR: 0.744, 95%CI: (0.634, 0.874), *P* < 0.001]; In different age groups, S-Klotho had a significantly greater effect on hyperuricemia in middle-aged people [age: 40–65 years, Q4 vs Q1, OR (95%CI): 0.69 (0.58, 0.82), *P* < 0.001; Age > 65 years: Q4 vs Q1, OR (95%CI): 0.72 (0.56, 0.92), *P* = 0.008)].When the level of S-Klotho was higher, the risk of hyperuricemia in men was lower than that in women [male: Q4 vs Q1, OR (95%CI): 0.67 (0.56, 0.81), *P* < 0.001; female: Q4 vs Q1 (95%CI):0.72 (0.58, 0.88),* P* < 0.001].

**Conclusions:**

In middle-aged and elderly individuals, plasma S-Klotho levels were inversely correlated with hyperuricemia, with a saturation effect. Given the limitations of the research results, the underlying mechanism between S-Klotho and hyperuricemia should be further explored.

## Introduction

Klotho, a protein known for its powerful anti-aging and neuroprotective properties, is considered the "star" protein of the longevity world. Klotho gene is located on human chromosome 13 [1], is primarily expressed in renal tubular epithelial cells, and encodes a unidirectional transmembrane protein, including two forms of circulating and membrane protein [2–4]. Among them, membrane proteins can be hydrolyzed to produce soluble circulating proteins (S-Klotho) [5], which are released into the serum and participate in various cell signaling pathways, thereby playing a role in the regulation of various regulating mineral metabolism [6–9]. Furthermore, S-Klotho deficiency has been confirmed to cause diseases affecting the cardiovascular system, nervous system, and blood system [10, 11], such as arteriosclerosis, osteoporosis, cognitive impairment, and the bone marrow hematopoietic function [12–14]. A study has shown that S-Klotho levels significantly decline in the aging brain tissue of mice and rhesus monkeys [15, 16]. Similar changes have been observed in human cerebrospinal fluid with aging, with Klotho gene expression declining with age or disease, causing pathological changes in the nervous system [12–14].

With population aging becoming the status quo of the demographic structure in many developed countries, it is extremely important to investigate whether plasma Klotho levels correlate with the prevalence of certain diseases in the middle-aged and elderly people. Over the past few decades, the prevalence of hyperuricemia has been high in middle-aged and elderly populations [17], and it is associated with a variety of comorbidities, such as hypertension, chronic kidney disease, metabolic syndrome, and cardiovascular disease [18]. By reducing the incidence of hyperuricemia among the middle-aged and elderly, the risk of these diseases will be delayed and the health and quality of life will be improved [28–30]. However, hyperuricemia is seldom recognized as a risk factor that needs to be independently controlled in the diagnosis and treatment of many middle-aged and elderly chronic diseases [19]. As of now, there has only been one study that has studied the relationship between serum uric acid and S-Klotho [32]. However, it does not clarified whether there was a correlation between S-Klotho and hyperuricemia in the middle-aged and elderly, since it studied the effect of serum uric acid on S-Klotho levels from the perspective of serum uric acid. Furthermore, whether there was a two-way effect between S-Klotho and serum uric acid levels also needs to be further clarified. Therefore, it is of great importance to determine whether S-Klotho levels affect the risk of hyperuricemia. In this study, data from the National Health and Nutrition Examination Survey (NHANES: 2007–2016) were used to examine the relationship between S-Klotho and the risk of hyperuricemia among middle-aged and elderly Americans.

## Materials and methods

### Study design and participants

**Fig. 1:**
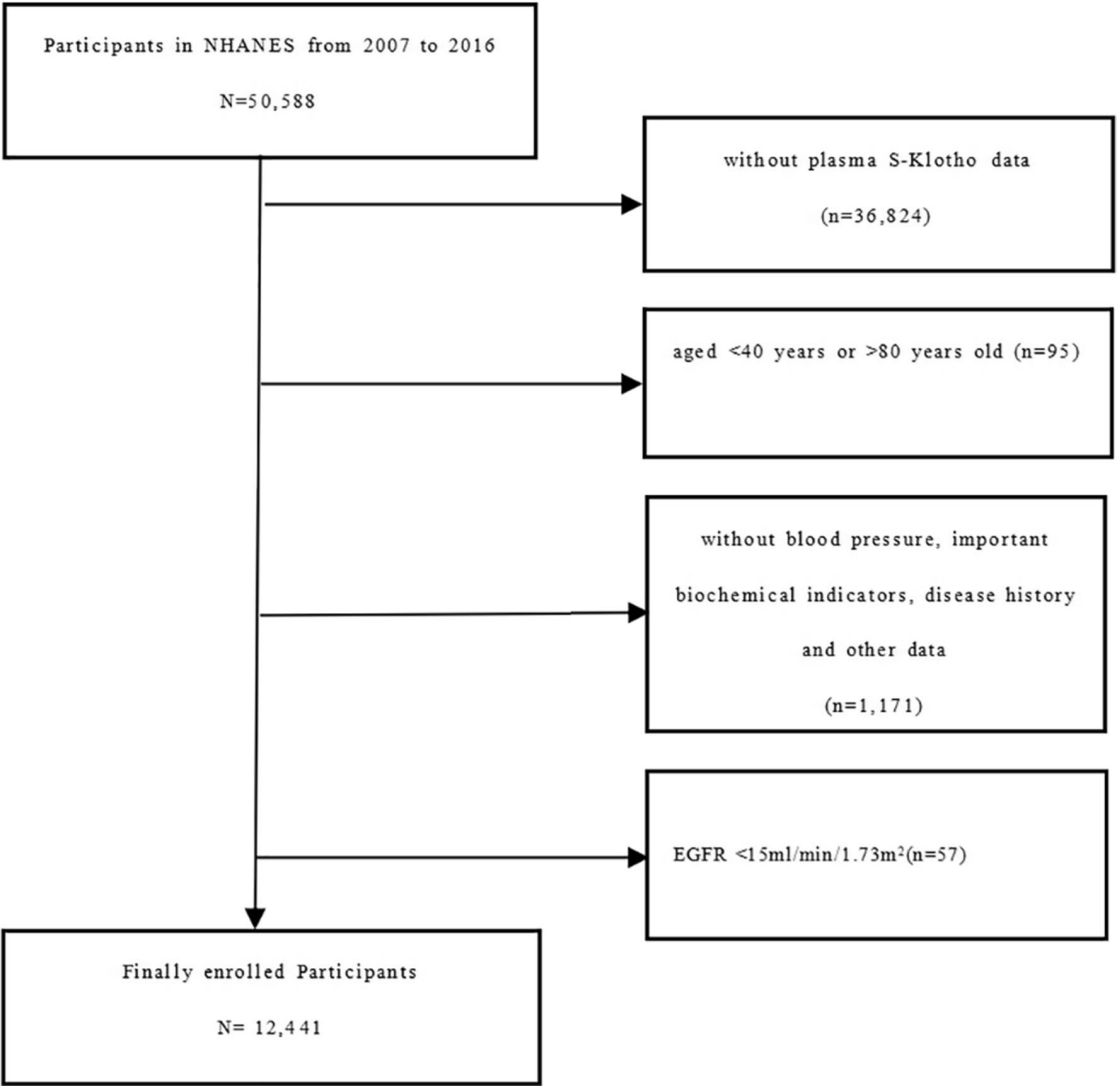
Study design and exclusion information flowchart

For this study, data were obtained from the National Health and Nutrition Examination Survey. NHANES is a large-scale cross-sectional survey of all levels of the U.S. population, which is composed of two parts: interviews and physical examinations. The survey includes such items as nutrition, health and sociology of American families, and gathers representative sample data to reflect the population as a whole. It has been conducted since 1990, and a nationally representative sample of about 5000 people is surveyed annually, every 2 years is a survey cycle. As part of the investigation, NHANES provided written informed consent to all individuals participating, which was reviewed by the Centers for Disease Control and Prevention (CDC) and the National Center for Health Statistics (NCHS). During the survey period from 2007 to 2016, the NHANES surveyed 50,588 people in five cycles (2007–2008, 2009–2010, 2011–2012, 2013–2014, 2015–2016). After a series of screenings, 12,441 people qualified for data analysis as subjects of this study. The population screening exclusion criteria are as follows: (1) subjects without plasma S-Klotho data (*n* = 36,824); (2) subjects aged < 40 years or > 80 years (*n* = 95); (3) subjects missing blood pressure, long-term health information, important biochemical indicators, disease history or other data (*n* = 1171); (4) eGFRCKD–EPI < 15 ml/min/1.73m^2^ (*n* = 57). Details of the study design and exclusions are provided in the flowchart (Fig. 1).

### Laboratory measurements

Blood samples were obtained from the venous blood of participants who had fasted for more than 9 h. The S-Klotho blood samples were stored in a (-80◦C) environment for data analysis in the University of Washington research laboratory. Researchers using a commercially available ELISA kit from Japan's IBL International, and using the sandwich ELISA to test S-Koltho after receiving the blood samples on dry ice [20]. To ensure the accuracy of the measured data, two quality control samples of low concentration and high concentration of Klotho were analyzed in duplicate in every ELISA board, and the final value was determined by calculating the average of the two results. The analysis results are automatically transmitted from the instrument to the laboratory Oracle management system, and the regional supervisor evaluates the results. Samples with repetitive results of more than 10% are marked as repetitive analysis. If the value of the quality control sample is not within the 2SD range of the specified value, the entire analysis run will be rejected and the sample analysis will be repeated. As a result of measuring and evaluating the samples of 114 obviously healthy individuals, S-Klotho ranged from 285.8 to 1638.6 pg/ml with a mean of 698.0 pg/ml. Biochemical indicators Blood samples are stored at (− 30 °C), and are analyzed by researchers using two methods: Beckman Synchron LX20 and Beckman UniCel® DxC800 Synchron. These biochemical indicators include urea, creatinine, serum uric acid, blood lipids, and fasting blood glucose levels. Hyperuricemia is defined as serum uric acid levels higher than or equal to 420 mmol/l in men and 360 mmol/l in women [21, 22].

### Covariates measurements

#### Blood pressure and body mass index

Mercury sphygmomanometers calibrated with Bowman meters were used to measure the blood pressure of all the subjects. Following 5 min of sitting, the trained examiner asked the subjects to take three consecutive blood pressure measurements on their right arm, with a 30 s interval between every measurement. Based on three blood pressure measurements, a mean blood pressure was calculated, and then either hypertensive or non-hypertensive classification was assigned. Hypertension was defined as systolic blood pressure greater than or equal to 140 mmHg, or diastolic blood pressure greater than or equal to 90 mmHg, or both. The body mass index was calculated based on height and weight, and the formula was: (Kg)/(m^2^).

#### Smoking, drinking and physical activity

A questionnaire survey was used to gather information on smoking and drinking for all participants. Using a computer-assisted personal interview (CAPI) system, the interviewer asked questions to the subjects, and the drinking and smoking conditions were assessed based on their responses. The specific evaluation criteria are as follows:

Question1. Smoked at least 100 cigarettes in life?

Answer No: means no smoking.

Yes: represents smoking or has ever smoked.

(1) How long since quit smoking cigarettes?

No: Delegate still smoking.

Yes: Quit smoking.

Question2. Had at least 12 alcohol drinks/1 year?

Answer Yes: drink a lot.

No: little or no alcohol consumption.

(1) Had at least 12 alcohol drinks/lifetime?

No: hardly drink alcohol.

Yes: occasional drinking.

Considering low physical activity is directly related to low levels of S-Klotho, especially in middle-aged and elderly people, therefore, we include the variable of physical activity (PA) in the final model [42, 43]. The physical activity data obtained from the physical activity questionnaire (PAQ) filled out by all participants, which asked whether they had engaged in vigorous-intensity sports, fitness, or recreational activities for at least 10 min, resulting in large increases in breathing or heart rate; and whether they were involved in moderate-intensity sports, fitness or recreational activities cause small increases in breathing or heart rate and was done for at least 10 min continuously.

#### Personal medical history

During home interviews, the NHANES interviewer collected the subject's personal medical history in the form of a questionnaire. As part of the interview, the subjects were asked whether they had been told by a doctor or other professional health personnel that they had congestive heart failure, coronary heart disease, stroke, and diabetes. Accordingly, if the subject answers yes to any of the above-mentioned diseases, he is required to provide the date and medication status of the first diagnosis of the disease, to better collect personal disease history data. Renal function was assessed by eGFR using the Chronic Kidney Disease–Epidemiology Collaboration (CKD–EPI) equation, and those with an EGFR < 15 ml/min/1.73 m^2^ were excluded based on the results of the calculation.

### Statistical analysis

The NHANES used a complex multistage probability sampling design to represent the health and nutrition status of the population at all levels within the United States, with oversampling in some subgroups. To avoid biased results in analysis and exaggeration of significance, the analyses were conducted in accordance with guidelines provided by the US Centers for Disease Control and Prevention and using the sample weights recommended by the guidelines. We calculated the sample weights of the two periods from 2007 to 2016, and completed the weight combination. The specific calculation formula is: MEC10YR = 1/5*WTMEC2YR. For continuous variables with a normal distribution, the data can be represented by the weighted mean ± SD; assuming the data have a skewed distribution, the weighted median is used to characterize the data mean, while the interquartile range describes the degree of dispersion; Comparing quantitative data between groups requires applying the Kruskal Wallis rank-sum test. The categorical variables were described by their rates or constituent ratios, and comparisons between groups were made with the chi-square test or Fisher's exact test. To test the correlation between the level of S-Klotho and the risk of hyperuricemia, a multiple logistic regression model was established based on the results of the univariate analysis, and the covariates with significant effects (*P* < 0.05) in the univariate analysis were included in the adjustment model.

The following models were developed: model 1, without adjusting for covariates; model 2, validated again after including age, sex, and race variables; model 3, with model 2 adjustment variables, as well as variables, such as blood pressure, smoking, BMI, urea, creatinine, triglyceride, high density cholesterol, low density cholesterol, fasting blood glucose, glycosylated hemoglobin, eGFRCKD–EPI, physical activity (PA), past disease history, etc. Considering that drinking may influence uric acid metabolism, drinking variables were included in the final model. Moreover, we conducted subgroup analyses by age and gender based on the model's final results, and the subgroup stratification variables were not included in their corresponding adjusted models. Finally, smooth curve fitting is used to show relationships between S-Klotho and the risk of hyperuricemia in middle-aged and elderly people. All data analysis was done by the statistical software package R (The R Foundation; http://www.r-project.org; version 3.5.3) and EmpowerStats (www.EmpowerStats.com; X&Y Solutions Inc.). *P* value of < 0.05 (double) was considered as statistically significant.

## Results

### Baseline characteristics of study participants

On the basis of plasma S-Klotho levels, 12,441 subjects (male: 48.70%) were divided into four groups: Q1: < 653.9 pg/ml, Q2: ≥ 653.9 to < 801.3 pg//ml, Q3: ≥ 801.3 to < 992.5 pg/ml, Q4: ≥ 992.5 pg/ml, and the study population's overall characteristics are given in Table 1. In general, compared with middle-aged and elderly individuals with lower plasma S-Klotho levels, people with higher plasma S-Klotho levels were more likely to be female, younger, and have hypertension, hyperuricemia, heart failure, coronary heart disease, stroke, and diabetes prevalence is lower. Moreover, they are mostly non-Hispanic white individuals. In terms of uric acid metabolism risk factors, middle-aged and elderly individuals with high plasma S-Klotho levels have lower urea, creatinine, total cholesterol, triglyceride, and low-density cholesterol levels, less smoking and drinking individuals, while their fasting blood glucose level and glycosylated hemoglobin level are higher (Tables 1, 2).

**Table 1:** Baseline characteristics of participants

Characteristic	Total population	S-Klotho levels quartiles, pg/ml				*P* value
		Q1	Q2	Q3	Q4	
	843.6 (833.6,853.6)	< 653.9	≥ 653.9 to < 801.3	≥ 801.3 to < 992.5	≥ 992.5	
Age	56.29 (55.99,56.60)	57.46 (57.01,57.91)	56.46 (55.92,57.00)	55.89 (55.48,56.31)	55.26 (54.77,55.75)	< 0.001
*Gender (%)*						< 0.001
Man	48.02 (47.08,48.96)	49.13 (46.88,51.38)	51.08 (48.96,53.18)	48.18 (45.99,50.37)	43.28 (41.04,45.54)	
Woman	51.98 (51.04,52.92)	50.87 (48.62,53.12)	48.92 (46.82,51.04)	51.82 (49.63,54.01)	56.72 (54.46,58.96)	
*Race/ethnicity (%)*						< 0.001
Mexican American	6.48	6.41	5.97	6.76	6.84	
Other Hispanic	4.55	3.95	4.26	4.59	5.45	
Non-Hispanic White	73.97	74.63	77.07	74.7	69.03	
Non-Hispanic Black	9.03	9.06	7.28	7.43	12.67	
Other Race	5.97	5.95	5.42	6.52	6.01	
*Blood pressure (%)*						0.002
Non-hypertension	58.25	54.91	59.08	60.62	58.23	
hypertension	41.75	45.09	40.92	39.38	41.77	
*Smoke (%)*						< 0.001
Don't smoke	51.39	47.16	50.11	52.07	56.54	
still smoking	18.42	20.92	19.55	17.23	15.82	
quit smoking	30.19	31.92	30.34	30.7	27.64	
*Alcohol*						< 0.001
Don't drink	10.55	8.87	10.91	9.91	12.6	
Drink a lot	77.47	80.49	78.57	77.43	73.12	
Drink occasionally	11.98	10.64	10.52	12.66	14.28	
*Physical activity (%)*						< 0.001
Vigorous recreational activities	15.06	16.7	18.59	20.48	19.8	
Moderate recreational activities	28.51	33.52	33.02	30.35	29.54	
Lack of recreational activities	56.43	49.78	48.39	49.18	50.66	
Body mass index (kg/m^2^)	29.57 (29.37,29.78)	29.67 (29.37,29.97)	29.63 (29.29,29.97)	29.63 (29.27,29.99)	29.34 (28.95,29.73)	0.489
Urea (mmol/l)	5.07 (5.01,5.13)	5.24 (5.15,5.33)	5.09 (5.00,5.17)	5.01 (4.92,5.11)	4.84 (4.74,4.94)	< 0.001
Creatinine (mmol/l)	79.69 (78.83,80.55)	81.91 (80.83,83.00)	79.13 (78.13,80.13)	77.51 (76.69,78.33)	75.00 (74.15,75.84)	< 0.001
Total cholesterol (mmol/l)	5.21 (5.17,5.24)	5.23 (5.16,5.30)	5.21 (5.15,5.26)	5.20 (5.14,5.26)	5.19 (5.13,5.25)	0.865
Triglyceride (mmol/l)	1.88 (1.83,1.92)	1.99 (1.87,2.12)	1.90 (1.83,1.97)	1.84 (1.79,1.90)	1.77 (1.70,1.84)	0.001
High density cholesterol (mmol/l)	1.40 (1.39,1.42)	1.41 (1.38,1.44)	1.38 (1.36,1.40)	1.40 (1.38,1.42)	1.42 (1.40,1.44)	0.042
Low density cholesterol (mmol/l)	3.43 (3.40,3.46)	3.42 (3.36,3.48)	3.45 (3.39,3.50)	3.43 (3.39,3.48)	3.42 (3.37,3.47)	0.921
*Hyperuricemia*						< 0.001
No	81.08 (79.81,82.28)	75.23 (73.03,77.30)	80.49 (78.29,82.52)	83.18 (81.19,85.01)	85.68 (83.55,87.57)	
Yes	18.92 (17.72,20.19)	24.77 (22.70,26.97)	19.51 (17.48,21.71)	16.82 (14.99,18.81)	14.32 (12.43,16.45)	
Fasting blood glucose (mmol/l)	5.79 (5.73,5.85)	5.64 (5.57,5.71)	5.70 (5.60,5.80)	5.77 (5.69,5.85)	6.07 (5.94,6.20)	< 0.001
Glycosylated haemoglobin (%)	5.80 (5.77,5.83)	5.7 (5.7, 5.8)	5.7 (5.7, 5.8)	5.7 (5.7, 5.8)	5.9 (5.9, 6.0)	< 0.001
*Heart failure (%)*						< 0.001
No	97.08 (96.73,97.39)	95.84 (94.99,96.56)	97.53 (96.86,98.06)	97.22 (96.51,97.79)	97.85 (97.20,98.35)	
Yes	2.92 (2.61,3.27)	4.16 (3.44,5.01)	2.47 (1.94,3.14)	2.78 (2.21,3.49)	2.15 (1.65,2.80)	
*Coronary heart disease (%)*						0.034
No	95.65 (95.11,96.13)	94.71 (93.71,95.56)	95.80 (94.78,96.62)	95.77 (94.82,96.55)	96.42 (95.30,97.28)	
Yes	4.35 (3.87,4.89)	5.29 (4.44,6.29)	4.20 (3.38,5.22)	4.23 (3.45,5.18)	3.58 (2.72,4.70)	
*Stroke (%)*						0.089
No	96.68 (96.31,97.01)	96.04 (95.00,96.88)	96.74 (95.97,97.36)	96.99 (96.31,97.55)	97.16 (96.37,97.78)	
Yes	3.32 (2.99,3.69)	3.96 (3.12,5.00)	3.26 (2.64,4.03)	3.01 (2.45,3.69)	2.84 (2.22,3.63)	
*Diabetes (%)*						0.159
No	86.63 (85.60,87.59)	85.77 (84.05,87.32)	86.77 (84.79,88.53)	88.07 (86.68,89.33)	86.23 (84.37,87.90)	
Yes	13.37 (12.41,14.40)	14.23 (12.68,15.95)	13.23 (11.47,15.21)	11.93 (10.67,13.32)	13.77 (12.10,15.63)	
eGFRCKD–EPI (ml/min/1.73m^2^)	85.73 (85.15,86.30)	82.70 (81.82,83.58)	85.68 (84.71,86.64)	86.79 (86.10,87.48)	88.61 (87.79,89.43)	< 0.001

Continuous variables are described by average ± standard deviation or median (quartile spacing); description of adoption rate and constituent ratio of classified variables

Urea, Creatinine, Total cholesterol, Triglyceride, High density cholesterol, Low density cholesterol, Fasting blood glucose: (mmol/l)

**Table 2:** Results of single factor analysis

	Univariate analysis for Hyperuricemia	
	OR (95%CI)	*P* value
Age	1.03 (1.02, 1.03)	< 0.001
Gender	0.89 (0.78, 1.02)	0.087
Race/ethnicity	1.38 (1.34, 1.68)	0.001
Blood pressure	2.73 (2.41, 3.09)	< 0.001
Smoke	1.41 (1.24, 1.62)	< 0.001
Alcohol	1.11 (0.94, 1.30)	0.219
Physical activity (%)	0.60 (0.52, 0.69)	< 0.001
Body mass index (kg/m^2^)	1.09 (1.08, 1.10)	< 0.001
Urea	1.30 (1.26, 1.33)	< 0.001
Creatinine	1.03 (1.02, 1.03)	< 0.001
Total cholesterol	1.04 (0.98, 1.09)	0.243
Triglyceride	1.13 (1.06, 1.21)	< 0.001
High density cholesterol	0.48 (0.40, 0.56)	< 0.001
Low density cholesterol	1.111 (1.04, 1.18)	0.002
S-Klotho	0.99 (0.98, 1.00)	< 0.001
Fasting blood glucose	1.06 (1.03, 1.09)	< 0.001
Glycosylated haemoglobin	1.14 (1.08, 1.20)	< 0.001
Heart failure	3.48 (2.69, 4.51)	< 0.001
Coronary heart disease	1.45 (1.11, 1.90)	0.007
Stroke	2.05 (1.59, 2.64)	< 0.001
Diabetes	1.82 (1.55, 2.13)	< 0.001
eGFRCKD–EPI (ml/min/1.73m^2^)	0.965 (0.961, 0.968)	< 0.001

### Association between S-Klotho and hyperuricemia

**Fig. 2:**
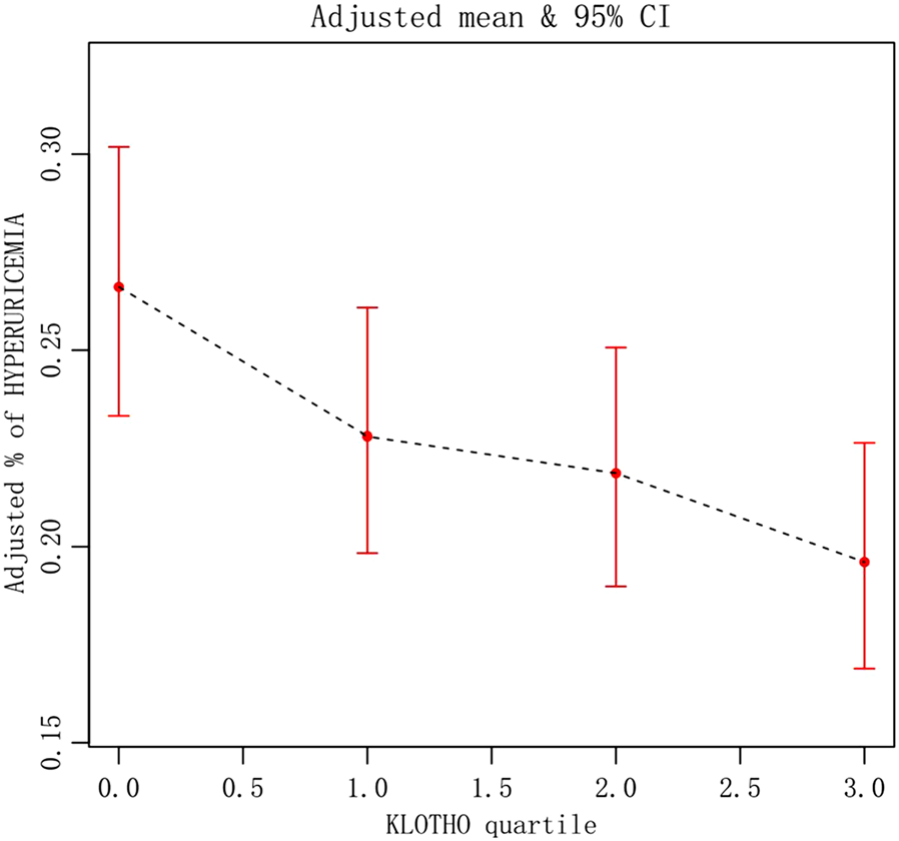
Relationship between S-Klotho and hyperuricemia in middle-aged and elderly people(S-Klotho quartiles)

**Fig. 3:**
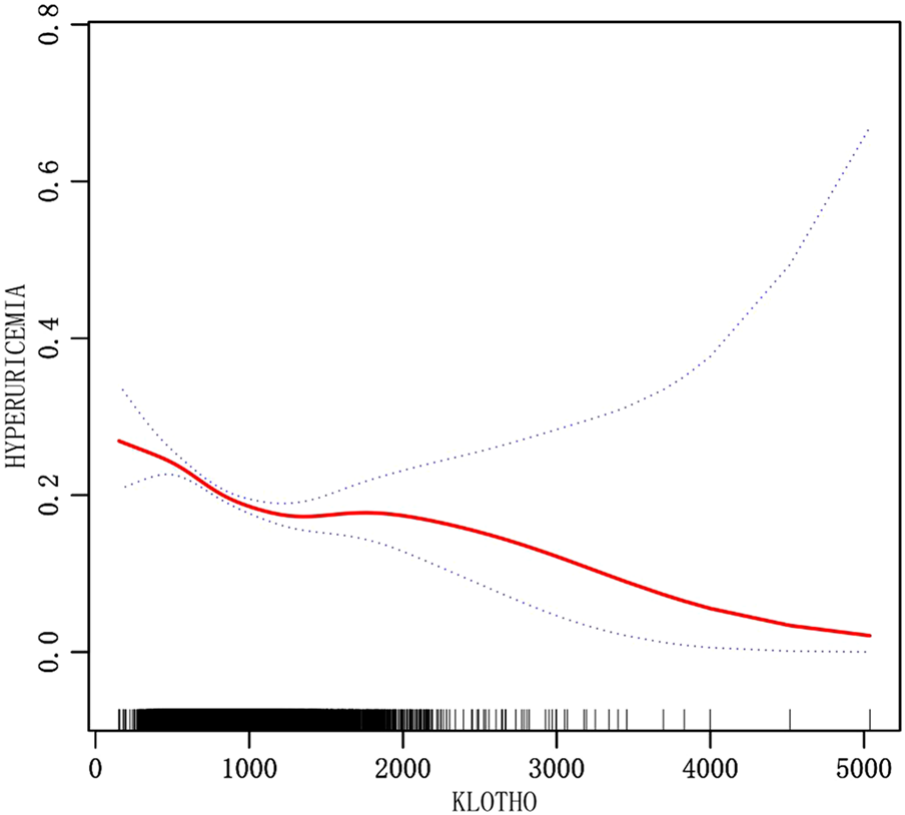
Relationship between S-Klotho and hyperuricemia in middle-aged and elderly people(smooth curve fitting)

**Fig. 4:**
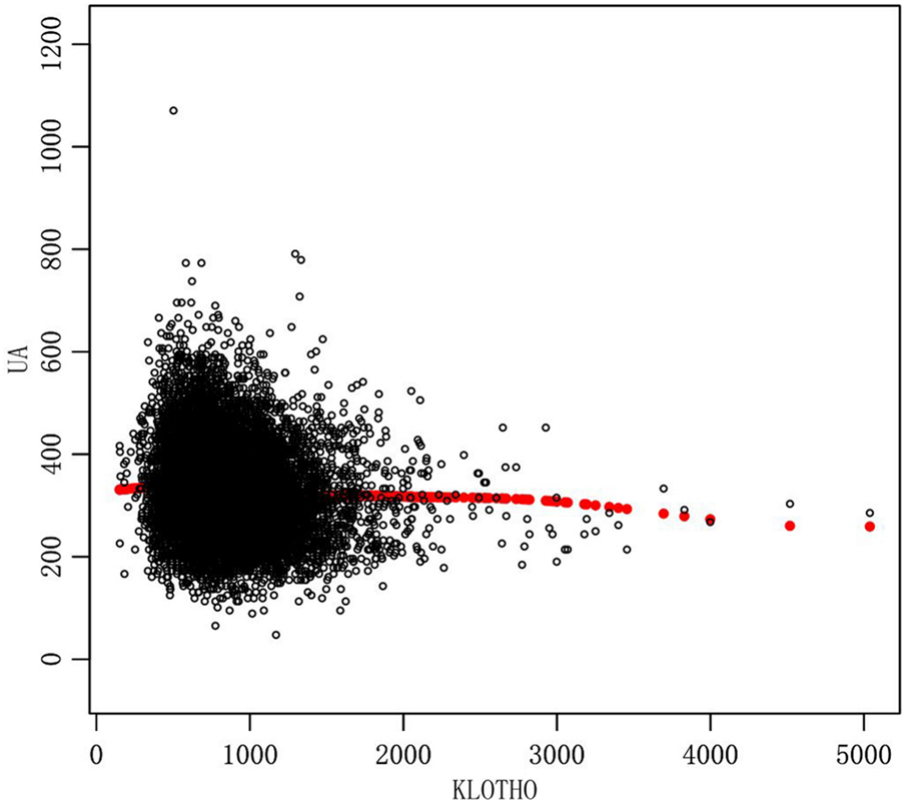
Relationship between S-Klotho and uric acid in middle-aged and elderly people(each black dot represents a sample)

**Fig. 5:**
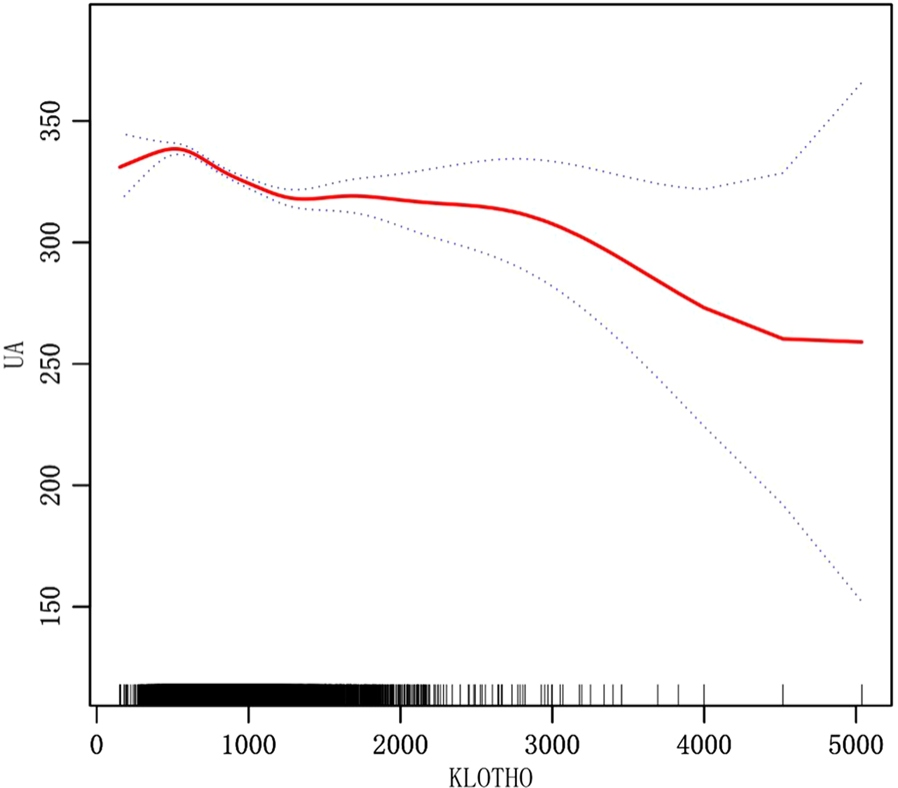
Relationship between S-Klotho and uric acid in middle-aged and elderly people

Based on multivariate logistic regression analysis, Table 3 summarized the findings of the relationship between plasma S-Klotho levels and hyperuricemia. In model 3, after having fully adjusted for age, sex, race, blood pressure, smoking, drinking, BMI, urea, creatinine, triglyceride, high density lipoprotein cholesterol (HDL-C), low density cholesterol, fasting blood glucose, glycosylated hemoglobin, eGFRCKD–EPI, physical activity (PA), history of heart failure, history of coronary heart disease, history of diabetes, and history of stroke, plasma S-Klotho was determined to be negatively related to hyperuricemia, and with higher levels of S-Klotho, the lower was the risk of hyperuricemia [Q4 vs Q1 (95%CI): 0.67 (0.59, 0.77), *P* < 0.001] (Table 3) (Fig. 2). As a result of smooth curve fitting, we observed a significant non-linear relationship between S-Klotho and hyperuricemia (*P* < 0.001) (Fig. 3). Therefore, we evaluated the threshold and saturation effects, and the results showed that the S-Klotho inflection point was 927.8 pg/ml (logarithmic likelihood ratio test = 0.002). On this basis, we performed piecewise Logistic regression analysis and found that higher plasma S-Klotho levels resulted in a 25.6% decreased prevalence of hyperuricemia when compared with those with lower plasma S-Klotho levels [OR: 0.744, 95%CI: (0.634, 0.874)]. As S-Klotho levels increased above 927.8 pg/ml, the association between S-Klotho levels and hyperuricemia risk in middle-aged and elderly individuals was no longer significant [OR: 0.891, 95%CI: (0.683, 1.163)] (Table 4). Thus, we believe that the piecewise logical regression model provides a better description of the relationship between plasma S-Klotho and hyperuricemia than the linear logical regression model (Figs. 4, 5).

**Table 3:** Relationship between S-Klotho pg/ml and hyperuricemia in middle-aged and elderly people

Outcome	Crude model		Model I		Model II	
	OR (95%CI)	*P* value	OR (95%CI)	*P* value	OR (95%CI)	*P* value
S-Klotho	0.99 (0.98, 1.00)	< 0.001	0.99 (0.98, 1.00)	< 0.001	1.00 (0.99, 1.00)	< 0.001
*S-Klotho (quartile)*						
Q1	Reference		Reference		Reference	
Q2	0.72 (0.63, 0.80)	< 0.001	0.74 (0.65, 0.83)	< 0.001	0.82 (0.72, 0.93)	0.002
Q3	0.62 (0.55, 0.70)	< 0.001	0.66 (0.58, 0.74)	< 0.001	0.77 (0.68, 0.88)	< 0.001
Q4	0.53 (0.47, 0.60)	< 0.001	0.53 (0.47, 0.61)	< 0.001	0.67 (0.59, 0.77)	< 0.001

Model 2: adjusted by age, gender, race

Model 3: with model 2 adjustment variables, as well as variables, such as blood pressure, smoking, BMI, urea, creatinine, triglyceride, high density cholesterol, low density cholesterol, fasting blood glucose, glycosylated hemoglobin, eGFRCKD–EPI, physical activity (PA), heart failure, coronary heart disease, stroke, diabetes. Considering alcohol may influence uric acid metabolism, drinking variable was included in the final model

**Table 4:** Saturation effect analysis between S-Klotho pg/ml and hyperuricemia using the piecewise logical regression model

Outcome	Crude model		Model II	
	OR (95%CI)	*P* value	OR (95%CI)	*P* value
S-Klotho ≤ 927.8 pg/ml				
*S-Klotho(quartile)*				
Q1	Reference		Reference	
Q2	0.824 (0.717, 0.947)	0.006	0.885 (0.759, 1.032)	0.119
Q3	0.657 (0.569, 0.758)	< 0.001	0.787 (0.671, 0.922)	0.003
Q4	0.603 (0.521, 0.698)	< 0.001	0.744 (0.634, 0.874)	< 0.001
S-Klotho > 927.8 pg/ml				
*S-Klotho(quartile)*				
Q1	Reference		Reference	
Q2	0.964 (0.766, 1.213)	0.754	0.962 (0.746, 1.240)	0.765
Q3	0.798 (0.630, 1.012)	0.063	0.799 (0.614, 1.041)	0.096
Q4	0.838 (0.663, 1.060)	0.143	0.891 (0.683, 1.163)	0.396

Age, gender, race, blood pressure, smoking, drinking, BMI, urea, creatinine, triglyceride, high density cholesterol, low density cholesterol, fasting blood glucose, glycosylated hemoglobin, eGFRCKD–EPI, physical activity (PA), heart failure, coronary heart disease, stroke, diabetes were adjusted

### Subgroup analysis

Due to the fact that uric acid metabolism may be affected by age and sex, we conducted a subgroup analysis based on the whole population to examine their effect on S-Klotheo and hyperuricemia risk in middle-aged and elderly people. The results showed that the relationship between middle-aged individuals was significantly higher than that of elderly individuals [age: 40–65 years, Q4 vs Q1, OR (95%CI): 0.69 (0.58, 0.82), *P* < 0.001; Age > 65 years: Q4 vs Q1, OR (95%CI): 0.72 (0.56, 0.92), *P* = 0.008)]. When S-Klotho levels are high, men are at lower risk of hyperuricemia than women [male: Q4 vs Q1, OR(95%CI): 0.67 (0.56, 0.81), *P* < 0.001; female: Q4 vs Q1 (95%CI):0.72 (0.58, 0.88),* P* < 0.001] (Table 5).

**Table 5:** Analysis of the association between S-Klotho pg/ml and hyperuricemia in subgroups

Stratified by gender	Crude model		Model I		Model II	
*Male*						
Q1	Reference		Reference		Reference	
Q2	0.72 (0.61, 0.85)	< 0.001	0.73 (0.61, 0.86)	< 0.001	0.80 (0.67, 0.95)	0.013
Q3	0.54 (0.45, 0.65)	< 0.001	0.55 (0.46, 0.66)	< 0.001	0.63 (0.52, 0.76)	< 0.001
Q4	0.56 (0.47, 0.67)	< 0.001	0.56 (0.47, 0.67)	< 0.001	0.67 (0.56, 0.81)	< 0.001
*Female*						
Q1	Reference		Reference		Reference	
Q2	0.73 (0.62, 0.87)	< 0.001	0.76 (0.64, 0.90)	0.001	0.90 (0.74, 1.09)	0.279
Q3	0.67 (0.57, 0.79)	< 0.001	0.70 (0.59, 0.83)	< 0.001	0.89 (0.73, 1.08)	0.226
Q4	0.51 (0.43, 0.61)	< 0.001	0.52 (0.43, 0.62)	< 0.001	0.72 (0.58, 0.88)	< 0.001
*Stratified by Age*						
***40–65 years***						
Q1	Reference		Reference		Reference	
Q2	0.77 (0.67, 0.89)	< 0.001	0.79 (0.68, 0.91) 0.0013	0.001	0.87 (0.74, 1.02)	0.081
Q3	0.65 (0.56, 0.76)	< 0.001	0.67 (0.58, 0.77)	< 0.001	0.78 (0.67, 0.92)	< 0.001
Q4	0.57 (0.49, 0.66)	< 0.001	0.56 (0.48, 0.65)	< 0.001	0.69 (0.58, 0.82)	< 0.001
*** > 65 years***						
Q1	Reference		Reference		Reference	
Q2	0.74 (0.60, 0.91)	0.005	0.76 (0.61, 0.94)	0.01	0.84 (0.66, 1.06)	0.145
Q3	0.58 (0.46, 0.72)	< 0.001	0.58 (0.46, 0.72)	< 0.001	0.68 (0.53, 0.87)	0.002
Q4	0.56 (0.45, 0.70)	< 0.001	0.53 (0.43, 0.67)	< 0.001	0.72 (0.56, 0.92)	0.008

## Discussion

This study was conducted with the primary objective of determining whether plasma S-klotho levels were independently related to hyperuricemia risk. As part of this study, we analyzed five NHANES cycle samples to reflect the overall middle-aged and elderly population in the United States. According to our results, there is a negative correlation between plasma S-Klotho and hyperuricemia in the entire sample population, and this relationship has a saturation effect, meaning the correlation between them ends when S-Klotho reaches 927.8 pg/ml. In addition, S-Klotho has a higher effect on uric acid metabolism in middle-aged people compared with elderly people. As compared to women, men who have higher levels of plasma S-Klotho are more protected against hyperuricemia. Over the past few decades, hyperuricemia has become a significantly prevalent condition among middle-aged and elderly individuals. There may be a variety of causes for this result including high purine diet, obesity, insufficient exercise, an unhealthy lifestyle, and others [23–25], a series of risk factors may contribute to this result. High serum uric acid levels are the main cause of gout. With a high uric acid level for long periods of time, urate crystals will be deposited in the joints and cause pain and inflammation. However, due to the phenomenon of asymptomatic hyperuricemia, high serum uric acid levels are rarely recognized and valued in middle-aged and elderly individuals [26]. According to current studies, hyperuricemia is associated with population mortality [27], affects the occurrence and progression of heart and kidney disease, and can be used as a predictor of obesity and metabolic syndrome [28–30]. A cohort study of people with heart failure found that hyperuricemia increases the risk of progression in patients with heart failure [31]. In our study, we found that the plasma levels of S-Klotho may have a protective effect against hyperuricemia in middle-aged and elderly people, so it is important to understand the effect of S-Klotho on hyperuricemia for middle-aged and elderly people, as well as clarify the potential benefits and risks of S-Klotho in these population groups.

Until now, only one study has reported the effect of serum uric acid level on S-Klotho [32]; however, it has not evaluated its effect on uric acid metabolism in middle-aged and elderly individuals from the perspective of plasma S-Klotho, or whether hyperuricemia is correlated with S-Klotho. Consequently, there was no evidence linking plasma S-Klotho levels to hyperuricemia in middle-aged and elderly individuals prior to this study. However, from the point of view of S-Klotho participating in cellular pathways and regulating metabolism in the body, the results we observed may be reasonable. The S-Klotho protein is a transmembrane protein that is a component of the endocrine fibroblast growth factor (FGF) receptor [33]. FGF23 is a phosphoric acid hormone, and its mechanism of action can regulate the increased uptake of inorganic phosphate by the body. When osteocytes secrete FGF23, the increase of FGF23 level can down-regulate the level of sodium-dependent phosphate cotransporter in proximal tubules. In addition, S-Klotho forms a receptor complex after binding to FGF23, which can play a physiological role in the parathyroid gland, down-regulates serum parathyroid hormone levels and activity, reduces calcium cycling, and increases urinary phosphate excretion [34–37]. Consequently, we hypothesize that the negative correlation between plasma S-Klotho levels and hyperuricemia may be owing to the FGF23/S-Klotho endocrine axis, and this may also explain the saturation effect between S-Klotho and hyperuricemia from another aspect.

In subgroup analyses, we observed a potential effect of age in the relationship between plasma S-Klotho and hyperuricemia, especially among middle-aged individuals (40–65 years). In this study, those with higher plasma S-Klotho levels tended to be younger and had lower levels of creatinine and urea, which is consistent with the protective effect of S-Klotho on the kidneys from renal damage. As a classic anti-aging gene, Klotho is mainly expressed in the kidney, and its expression level is negatively correlated with age. Current studies have confirmed that dysregulation of the FGF23-Klotho signaling pathway is significantly associated with the occurrence and progression of CKD, and leads to hyperphosphatemia and endothelial dysfunction [38]; in a mouse model of S-Klotho deficiency, the researchers also found evidence of renal impairment due to impaired urinary phosphate excretion [39]. Consequently, on one hand, the amount of expression of S-Klotho decreases with advancing age, and on the other hand, the elderly have a worse kidney function and a lower urate excretion as compared with the middle-aged population, which is prone to hyperuricemia. Therefore, the differential effects of S-Klotho observed in different age groups may be the result of comprehensive factors.

To our knowledge, this is the first study to examine the association between plasma S-Klotho and hyperuricemia using population samples from NHANES. Considering that we included multi-ethnic populations and conducted the analysis in line with guideline recommendations, our results might be closer to what actually occurs in the U.S. population when it comes to the relationship between S-Klotho and hyperuricemia. Furthermore, we performed a subgroup analysis of these data and found a saturation effect between S-Klotho and hyperuricemia, which represents the strength of this study. However, since this study is a cross-sectional survey, we cannot give an accurate causal relationship between hyperuricemia and plasma S-Klotho. In addition, the data on gout drug treatment in the NHANES were incomplete; therefore, we ultimately decided not to add the gout drug variable after careful consideration to avoid errors caused by missing data bias on the outcome. It cannot be ruled out that they may have an impact on the final result as confounding factors. In addition, since the study found that S-Klotho may have a circadian rhythm and temporal variation [40, 41], there may be potential interference with the final results due to the different blood collection times from the subjects. This aspect needs to be further explored and clarified in future studies.

## Conclusions

In summary, our results demonstrate that plasma S-Klotho correlates negatively with hyperuricemia in middle-aged and elderly individuals, and there is a saturation effect. When S-Klotho > 927.8 pg/ml, the correlation between them is no longer significant. Furthermore, compared with elderly individuals, S-Klotho has a greater impact on the risk of hyperuricemia in middle-aged individuals; when the plasma level of S-Klotho is higher, the risk of hyperuricemia is lower in men than that in women.

## Data Availability

Publicly available data sets were analyzed in this study. These data can be found here: https://wwwn.cdc.gov/nchs/nhanes/Default.aspx.
